# Prevalence of Cooking with Polluting Fuels and Association with Elevated Blood Pressure Among Adults in Port au Prince, Haiti: A Cross-Sectional Analysis

**DOI:** 10.5334/gh.1405

**Published:** 2025-02-28

**Authors:** Rehana Rasul, Rodney Sufra, Marie Christine Jean Pierre, Reichling St. Sauveur, Vanessa Rouzier, Joseph Inddy, Erline Hilaire, Fabiola Preval, Lily D. Yan, Nour Mourra, Anju Ogyu, Daniella M. Pierre, Jean William Pape, Denis Nash, Margaret L. McNairy

**Affiliations:** 1 Department of Epidemiology and Biostatistics, Graduate School of Public Health and Health Policy, City University of New York, NY 10027, USA; 2 Institute of Implementation Science in Population Health, City University of New York, NY 10027, USA; 3 Haitian Group for the Study of Kaposi’s Sarcoma and Opportunistic Infections (GHESKIO), 33 Boulevard Harry Truman, Port-au-Prince 6110, Haiti; 4 Center for Global Health Weill Cornell Medicine New York NY, USA; 5 Center for Global Health, Weill Cornell Medicine, 402 East 67th Street, NY 10065, USA; 6 Center for Global Health, Weill Cornell Medicine, 402 East 67th Street, 10065, USA; 7 Division of General Internal Medicine, Department of Medicine, Weill Cornell Medicine, 525 East 68th Street, Box 331, NY, 10065, USA

**Keywords:** polluting, clean fuel, fuel, cookstove, hypertension, cardiovascular

## Abstract

**Background:**

Cooking with polluting fuels is common in low- and middle-income countries and may impact blood pressure, yet data on this association in urban Haiti is limited. This study describes the prevalence of polluting fuel use and indoor cooking, evaluates their associations with blood pressure, and evaluates whether effects are heterogeneous by sex in urban Haiti.

**Methods:**

Using cross-sectional data from the Haiti Cardiovascular Disease Cohort study, prevalence of polluting fuel use and indoor cooking was estimated. The associations between polluting fuel use and indoor cooking with systolic blood pressure (SBP), diastolic blood pressure (DBP) and hypertension (HTN) (mean SBP ≥140 mmHg or mean DBP ≥90 mmHg) were estimated using generalized estimating equations. The interaction between polluting fuel use and sex was also evaluated.

**Results:**

Among 2,931 participants, 58.2% were female and the mean age was 42.0 (SD = 15.9) years. The majority (88.2%) primarily cooked with polluting fuels. Polluting vs clean fuel users tended to have less than a high school education (38.0% vs 22.8%), earn ≤ 1 USD/day (70.5% vs 67.4%), and have high food insecurity (85.0% vs 64.3%). Polluting vs clean fuel users had similar HTN prevalence (adjusted prevalence ratio [aPR] = 0.94, 95% CI: 0.80, 1.10). Average SBP was similar for women (0.99 mmHg, 95% CI: –1.46, 3.44) and lower for men (–4.80 mmHg, 95% CI: –8.24, –1.37) who used polluting vs clean fuels. Cooking indoors vs outdoors was associated with higher HTN prevalence (aPR = 1.12, 95% CI: 1.00, 1.25) and higher average SBP (1.67 mmHg, 95% CI: 0.15, 3.20).

**Conclusions:**

This study demonstrates that most Haitians in Port-au-Prince cook with polluting fuels and often indoors. Those with higher poverty are more exposed, with mixed results in their association with blood pressure. Longitudinal studies are needed to clarify causal relationships and inform interventions promoting clean fuel use. (ClinicalTrials.gov Identifier: NCT03892265).

## Introduction

Over 2 billion people worldwide primarily relied on polluting fuels for cooking, and an estimated 78% were from low-and-middle income countries (LMIC) in Africa and Haiti in 2021 (1). Cooking with polluting fuels is a major contributor to household air pollution (HAP), which may be generated in or around the home (2). HAP is estimated to cause 3.2 million deaths annually (1,3), with 55% of these deaths due to cardiovascular diseases (CVD) (4). This may be due to its effect on hypertension (HTN), which had an estimated prevalence of 17.5–34.0% in LMICs in 2019 and contributed to 2.6 times more deaths in these countries compared to high income countries (5). Blood pressure is directly and indirectly affected by burning polluting fuels like biomass, kerosene and charcoal (6,7,8). They produce products of incomplete combustion, which penetrate deep into the terminal alveoli in the lungs and can easily enter the bloodstream. These directly activate inflammatory responses and central sympathetic mechanisms (6,7,8). They indirectly cause inflammation by forming free radicals and phospholipids (6,7,8). These pathways cause endothelial dysfunction and vasoconstriction (6,7,8). The concentration of these pollutants are correlated with health risk and can vary due to factors including room volume and air exchange rate (2,9). Cooking indoors compared to in semi-outdoor or outdoor areas may increase HAP concentration due to these factors, but air exchange is bidirectional and the effect of cooking location on health outcomes is unclear (2,10).

Haiti has both high utilization of polluting fuels for cooking based on the Haiti Demographic and Health Surveys 2016–2017 and demographic and market surveys (11,12,13). Modeling data suggests that HAP is the second leading risk factor of disability-adjusted life year reduction in this country (14). Its age-adjusted HTN prevalence is 28.5% and has been increasing (3,14,15,16,17). It is unclear whether cooking with polluting fuels is contributing to this burden. Studies evaluating the effect of polluting fuels on elevated blood pressure have been mixed, likely due to variations in study design, sample size, region, different cooking fuel comparisons, type of stove, and participant selection (18,19,20). Observational studies suggest that burning solid fuels may increase HTN risk by 50% (18,20). Some interventional studies found decreased systolic blood pressure (SBP), while others found decreased diastolic blood pressure (DBP) when using cleaner improved cookstoves compared to traditional stoves (19). In Haiti, studies on polluting fuels are sparse. One study demonstrated that cooking fuel type was associated with HAP pollutants among women in rural areas of Haiti (21). However, they did not compare polluting vs clean fuels or link these exposures to elevated blood pressure. Further, strategies to reduce HAP require understanding whether groups at higher risk are differentially impacted and whether conditions affecting HAP concentrations play a role. Women are usually more exposed to higher HAP exposure (2,9,22) as the primary household cooks, and in Haiti, HTN prevalence is also disproportionately higher among women compared to men (3,15–16). Further, indoor cooking is high in this region (21), but it is unclear whether this affects blood pressure. Using the community-based Haiti CVD Cohort Study of urban Haitian residents (23), this study aims to describe the prevalence of polluting fuel use for cooking and indoor cooking, evaluate their association with elevated blood pressure outcomes, and evaluate whether any observed effects are heterogeneous by sex.

## Methods

### Study setting and population

The Haiti CVD Cohort Study is a community-based longitudinal cohort of Port-au-Prince residents, many of whom live in urban slum communities (23). It was conducted by the Groupe d’Haitien d’Etude du Sarcome de Kaposi et des Infections Opportunistes (GHESKIO), a public health clinic established in 1982 in downtown Port-au-Prince. Participants were selected using multistage random sampling with GPS waypoints within census blocks (23). Eligibility criteria included being ≥18 years old and residing in Port-au-Prince without plans to move in the next 24 months. Exclusion criteria included indication of serious medical conditions, cognitive impairment, or inability to speak French or Haitian Creole. Enrollment occurred from March 2019 to August 2021 and included 3,005 participants. Participants were given baseline survey questions regarding sociodemographics, household measures, and neighborhood characteristics. Anthropometric and lab measures were collected by providers at the GHESKIO clinic. The study was approved by the Institutional Review Board of Weill Cornell Medicine and GHESKIO (1803019037). Informed consent was obtained from all subjects involved in the study. For this study, a cross-sectional analysis of baseline data from the Haiti CVD Cohort Study was conducted. After excluding participants who were pregnant (n = 33) or indicated that no food was prepared in the home (n = 45), the study sample comprised 2,931 participants (Supplementary Figure 1).

### Cooking characteristics

Household cooking characteristics included primary cooking fuel type, location, and ventilation use, and were reported by the primary member of the household. Cooking fuel type was classified as polluting (charcoal, wood, agricultural residue, or kerosene) or clean (propane or electricity) fuels in accordance with the World Health Organization (WHO) definition (2). Cooking location was defined as indoors if cooking occurred in the home or in a separate building, and outdoors if cooking occurred outside the house. If indoors, ventilation was indicated if the cooking area was near a window or door. Being a primary cook (yes/no) was also indicated for those who worked as a cook or a food vendor or were the main cook for their household.

### Blood pressure outcomes

Blood pressure (BP) was measured at the GHESKIO clinic by a trained field worker under the supervision of a registered nurse and measured using a semi-automated oscillometric BP machine (Omron HEM 907) in accordance with WHO guidelines (24). BP outcomes were HTN, continuous SBP, and continuous DBP. HTN was defined as having a mean SBP ≥140 mmHg and/or a mean DBP ≥90 mmHg based on the average of the last two of three measurements, or self-reported indication of antihypertensive medication use. For description, prehypertension (preHTN) was also defined as mean SBP readings between 120 and 139 mmHg and/or mean DBP readings between 80 and 89 mmHg.

### Other study variables

Sociodemographic, anthropometric, and health behavior questions were from standardized WHO STEPwise Approach to NCD Risk Factor Surveillance instruments (24). Sociodemographic variables included age in years, sex, education (less than high school (HS) education, HS education or higher), income (<1 USD/day, 1–10 USD/day, >10 USD/day), and household size (1–3, ≥4 persons). Household food insecurity (no or low, moderate, high) was also asked using questions from the Six-Item Short Form of the Household Food Security Scale (25–26). Health behaviors included tobacco smoking (current or former/never) based on questions about past and current use of tobacco products, alcohol use (<1 drink a day, ≥1 drink a day) based on past 12-month usage, and physical activity (moderate-high/low) based on questions on physical activity duration and frequency (e.g., manual labor, sports, fitness, recreational activity) which increased breathing or heart rate for ≥75 minutes per week. Body mass index (BMI) in kg/m^2^ was also calculated using height and weight measured at the GHESKIO clinic and categorized as underweight/normal (<25 kg/m^2^), overweight (25–29 kg/m^2^), and obese (≥30 kg/m^2^). Season of enrollment, which may affect cooking fuel type and location, was classified as rainy (April–June, August–November) and dry otherwise. Diabetes was indicated if fasting glucose ≥126 mg/dL or non-fasting glucose ≥200 mg/dL based on WHO/IDF definitions (27), self-reported indication of diabetes medication use, or diabetes diagnosis from a study physician. Dyslipidemia was indicated if total cholesterol ≥240 mg/dL, low-density lipoprotein level ≥160 mg/dL, or indication of use of statins (28). Kidney disease was indicated if estimated glomerular filtration rate (eGFR) <60 mL/min/1.73 m^2^ or urine albumin to creatinine ratio ≥30 mg/g according to Kidney Disease: Improving Global Outcomes guidelines (29), with eGFR calculated using the 2021 Chronic Kidney Disease Epidemiology Collaboration creatinine-based equation without ethnic factor (30).

### Statistical analyses

Distributions of participant characteristics were described by cooking fuel type. The association between cooking fuel type and the outcome of HTN was evaluated using unadjusted and adjusted multivariable generalized estimating equations (GEE) with a Poisson distribution to account for clustering of participants by household. A directed acyclical graph was used to determine the covariates needed to adjust for confounding (Supplementary Figure 2). Covariates included age, sex, poverty, education, household food insecurity, and household size. To account for missing data from covariates (n = 119, 4.1%), the data were first imputed 10 times to account for uncertainty due to missing values using the R package *mitml* (31). The imputations were based on all covariates and included each outcome, smoking status, BMI category, and BP treatment as auxiliary variables. This method assumes that data are missing at random, conditional on the variables included (32). Then the model was performed on each imputed dataset and Rubin’s rules were used to pool estimates and standard errors (32). Cooking location likely mediates the relationship between cooking fuel type and BP outcomes since cooking location may be chosen based on fuel type, and thus not adjusted for in this model. The relationship between cooking location and HTN was separately evaluated using GEE given the relevance of location in affecting HAP exposure, adjusting for sociodemographics, as well as cooking fuel type (33). The associations between cooking fuel type and cooking location and SBP and DBP were evaluated using multivariable linear GEE models, adjusting for the same covariates listed for each exposure. To evaluate possible differential effects by sex, a separate model was conducted including an interaction between cooking fuel type and sex. Estimated population averaged adjusted prevalence ratios (aPR) for Poisson regressions and adjusted beta coefficients (adjB) for linear regressions and corresponding 95% confidence intervals (CI) based on robust standard errors were reported. Analyses were conducted using R, version 4.3.1 and utilized package *ggplot2* for graphics (34).

### Sensitivity analyses

Several post hoc analyses were performed to check the sensitivity of the main results to different factors affecting exposures and outcomes. First, models were performed again including only primary cooks and those aged ≥35 years, since these groups were expected to have higher duration and intensity of polluting fuel exposure. For the analysis on primary cooks only, since very few participants were male cooks, a model including the interaction between cooking fuel type and sex was not performed. Second, models were performed on participants who were not on BP medications to account for its influence on BP measures. Third, models were performed with smoking and BMI status as covariates, which as risk factors for BP, may affect the precision of results. These analyses were exploratory, and P values were not adjusted for multiple testing.

## Results

**Figure 1:**
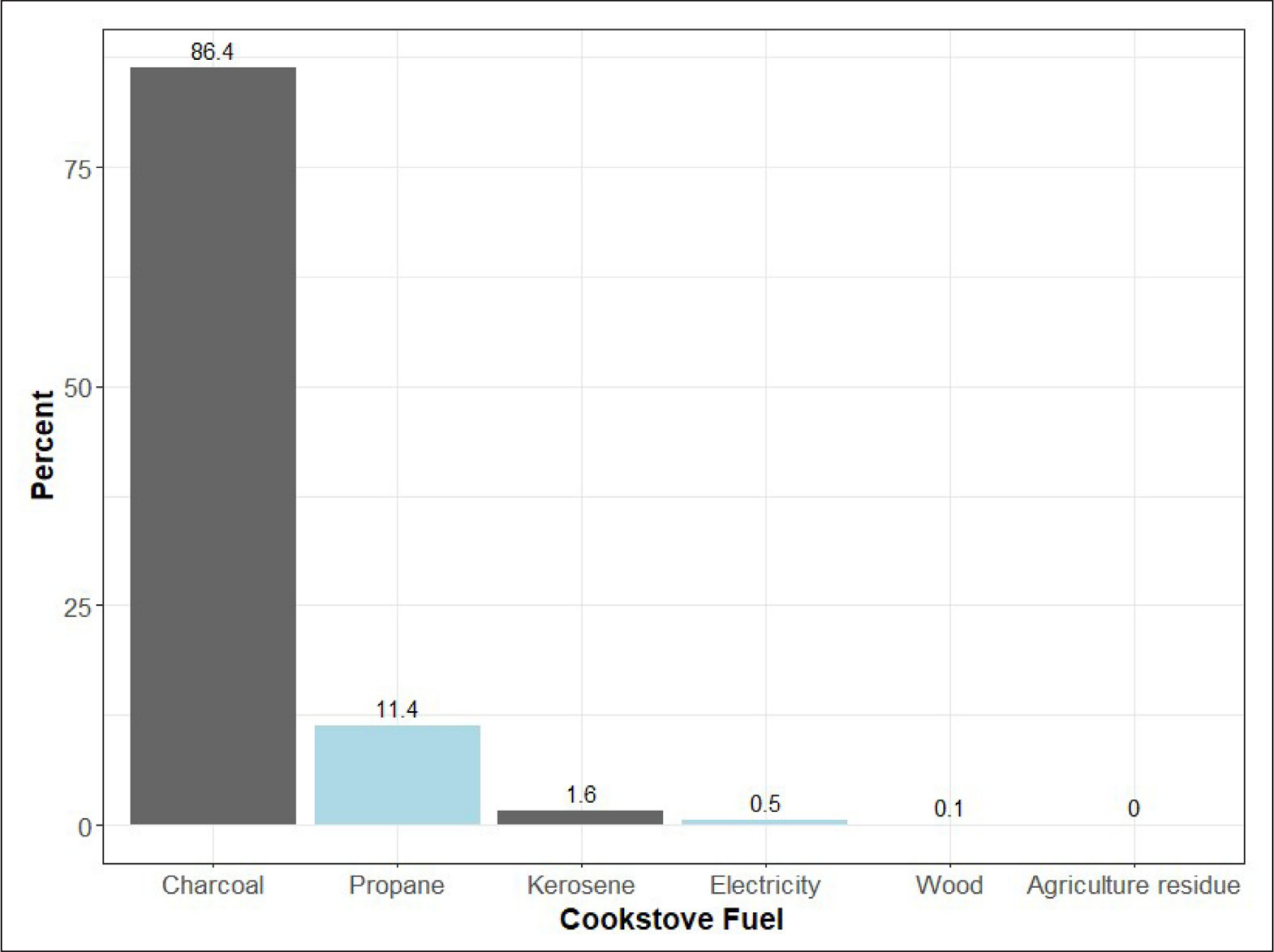
Type of cooking fuel. Dark gray and light blue bars represent polluting and clean fuel, respectively.

Among 2,931 participants, 58.2% were female and the mean age was 42.0 (SD = 15.9) years old (Table 1). Polluting fuels were the primary cooking fuels in homes for 88.2% of participants, with the majority (98.0%) using charcoal (Figure 1). Among the clean fuel users (11.8%), propane gas was the predominant fuel (96.0%). Cooking occurred mostly indoors (66.9%) for all participants. The prevalence of indoor cooking was lower among those who used polluting fuels compared to participants using clean fuels. When cooking indoors, ventilation was nearby 96.0% of the time. The proportion of primary cooks was a little higher among those using clean fuels. Participants exposed to polluting compared to clean fuels had similar mean (SD) age (42.3 (15.9) vs 40.4 (15.1) years) (Table 1). They tended to have lower socioeconomic status with respect to HS education, income, and food insecurity compared to participants using clean fuels. Physical activity was slightly higher among those using polluting fuels, whereas overweight or obese status was lower among those using polluting compared to clean fuels.

**Table 1:** Prevalence of study characteristics compared by cooking fuel type. SD = standard deviation; CVD = cardiovascular disease; HS = high school.


	OVERALL	CLEAN FUELS	POLLUTING FUELS

	N = 2931 (100%)	N = 347 (11.8%)	N = 2584 (88.2%)

***Demographics***			

**Age in years**, Mean (SD)	42.03 (15.85)	40.40 (15.07)	42.25 (15.94)

**Age group, years**			

18–29	852 (29.1%)	106 (30.5%)	746 (28.9%)

30–39	552 (18.8%)	85 (24.5%)	467 (18.1%)

40–49	525 (17.9%)	56 (16.1%)	469 (18.2%)

50–59	496 (16.9%)	50 (14.4%)	446 (17.3%)

60+	506 (17.3%)	50 (14.4%)	456 (17.6%)

**Sex**			

Male	1226 (41.8%)	127 (36.6%)	1099 (42.5%)

Female	1705 (58.2%)	220 (63.4%)	1485 (57.5%)

**Education**			

<HS	1059 (36.2%)	79 (22.8%)	980 (38.0%)

≥HS	1865 (63.8%)	268 (77.2%)	1597 (62.0%)

Missing	7	0	7

**Income**			

≤ $1 USD/day	2052 (70.2%)	234 (67.4%)	1818 (70.5%)

$1–10 USD/day	354 (12.1%)	38 (11.0%)	316 (12.3%)

>$10 USD/day	518 (17.7%)	75 (21.6%)	443 (17.2%)

(Missing)	7	0	7

**Household size**			

1–3	1639 (55.9%)	160 (46.1%)	1479 (57.2%)

4+	1292 (44.1%)	187 (53.9%)	1105 (42.8%)

**Food insecurity**			

No/Low FIS	53 (1.8%)	22 (6.5%)	31 (1.2%)

Moderate FIS	448 (15.6%)	98 (29.2%)	350 (13.8%)

High FIS	2371 (82.6%)	216 (64.3%)	2155 (85.0%)

Missing	59	11	48

**Season**			

Dry	1,351 (46.1%)	168 (48.4%)	1,183 (45.8%)

Rainy	1,580 (53.9%)	179 (51.6%)	1,401 (54.2%)

***Cooking related***			

**Cooking location**			

Inside home	509 (17.4%)	79 (22.8%)	430 (16.6%)

Outside	967 (33.0%)	38 (11.0%)	929 (36.0%)

In a separate building	1451 (49.5%)	230 (66.3%)	1221 (47.3%)

Missing	4 (0.1%)	0 (0.0%)	4 (0.2%)

**Cook status**			

Not primary cook	1818 (62.2%)	208 (59.9%)	1610 (62.5%)

Primary Cook	1106 (37.8%)	139 (40.1%)	967 (37.5%)

Missing	7	0	7

***Health behaviors***			

**Current smoking**			

Current/Former	216 (7.4%)	19 (5.5%)	197 (7.7%)

Never	2,696 (92.6%)	325 (94.5%)	2371 (92.3%)

(Missing)	19	3	16

**Alcohol use**			

<1 drink/day	2,807 (96.3%)	333 (96.2%)	2474 (96.3%)

≥1 drink/day	109 (3.7%)	13 (3.8%)	96 (3.7%)

Missing	15	1	14

**Physical activity**			

Moderate–high	1,442 (49.4%)	159 (45.8%)	1283 (49.9%)

Low	1489 (50.6%)	188 (54.2%)	1289 (50.1%)

Missing	12	0	12

***CVD Risk factors***			

**Body mass index categories**			

Underweight/Normal weight	1,641 (56.5%)	143 (41.6%)	1498 (58.5%)

Overweight	765 (26.3%)	112 (32.6%)	653 (25.5%)

Obese	500 (17.2%)	89 (25.9%)	411 (16.0%)

Missing	25	3	22

**Diabetes**			

Yes	154 (5.4%)	19 (5.5%)	135 (5.3%)

No	2,691 (94.59%)	317 (91.4%)	2,374 (91.9%)

Missing	86 (2.9%)	11 (3.2%)	75 (2.9%)

**Dyslipidemia**			

Yes	364 (12.4%)	37 (10.7%)	327 (12.7%)

No	2560 (87.6%)	309 (89.3%)	2251 (87.3%)

Missing	7 (0.2%)	1 (0.3%)	6 (0.2%)

**Kidney Disease**			

Yes	248 (8.7%)	26 (7.7%)	222 (8.8%)

No	2662 (91.3%)	318 (92.3%)	2344 (91.2%)

Missing	21 (0.7%)	3 (0.9%)	18 (0.7%)

**BP Treatment**			

Yes	348 (12.0%)	47 (13.7%)	301 (11.7%)

No	2562 (88.0%)	297 (86.3%)	2265 (88.3%)

Missing	21 (0.7%)	3 (0.9%)	18 (0.7%)


The prevalence of HTN and pre-HTN were 29.4% and 21.7% respectively (Table 2). HTN prevalence (29.6% vs 27.4%) and mean SBP and DBP were similar by cooking fuel type. Interestingly, the prevalence of preHTN was slightly lower (21.5% vs 23.3%) among those using polluting vs clean fuels. For females, higher HTN prevalence and DBP were found among polluting compared to clean fuel users. However, for males, pre-HTN was lower among those using polluting compared to clean fuels.

**Table 2:** Prevalence of blood pressure outcomes compared by cooking fuel type and sex. BP = blood pressure, HTN = hypertension, SBP = systolic blood pressure, DBP = diastolic blood pressure, SD = standard deviation.


	TOTAL			MALE		FEMALE	

	OVERALL	CLEAN FUELS	POLLUTING FUELS	CLEAN FUELS	POLLUTING FUELS	CLEAN FUELS	POLLUTING FUELS
		
	N = 2931	N = 347	N = 2584	N = 127	N = 1099	N = 220	N = 1485

**Blood pressure categories**							

Normal	1,426 (49.0%)	170 (49.4%)	1,256 (48.9%)	50 (39.4%)	532 (48.7%)	120 (55.3%)	724 (49.1%)

PreHTN	631 (21.7%)	80 (23.3%)	551 (21.5%)	44 (34.6%)	269 (24.6%)	36 (16.6%)	282 (19.1%)

HTN	854 (29.4%)	94 (27.4%)	760 (29.6%)	33 (26.2%)	292 (26.7%)	61 (28.1%)	468 (31.8%)

**SBP (mmHg)**, Mean (SD)	124.41 (23.89)	123.09 (22.17)	124.59 (24.11)	127.87 (20.90)	125.64 (22.90)	120.31 (22.45)	123.81 (24.94)

**DBP (mmHg)**, Mean (SD)	74.11 (15.51)	73.73 (14.85)	74.17 (15.60)	72.83 (15.77)	72.54 (15.85)	74.24 (14.31)	75.37 (15.31)


From the multivariable models (Table 3), there was no evidence of a difference in the prevalence of HTN among those who used polluting compared to clean fuels (aPR = 0.94, 95% CI: 0.80, 1.10). Cooking fuel type was also not associated with SBP or DBP. However, sex modified the relationship between cooking fuel type and SBP. For men, average SBP was 4.80 mmHg lower (95% CI: –8.24, –1.37) among polluting fuel users compared to clean fuel users, whereas for women, there was no difference in average SBP by cooking fuel type (adjB = 0.99, 95% CI: –1.46, 3.44). There was no evidence that sex modified the relationship between cooking fuel type and HTN or DBP. Cooking indoors vs outdoors was associated with 12% (aPR: 1.12, 95% CI: 1.00, 1.25) higher prevalence of HTN and 1.67 mmHg higher (95% CI: 0.15, 3.20) average SBP, while adjusting for cooking fuel type and sociodemographics. Cooking location was not associated with DBP. The post hoc analyses yielded similar results to the main analysis (Supplementary Table 1). It is noted that for participants not on BP medications, the effect of indoor vs outdoor cooking on HTN and SBP were similar, but confidence intervals included the null value (Supplementary Table 1).

**Table 3:** Results of the unadjusted and adjusted associations of cooking fuel type and cooking location on blood pressure outcomes. PR = prevalence ratio, CI = confidence interval, SBP = systolic blood pressure, DBP = diastolic blood pressure.


EFFECT	HYPERTENSION	SBP	DBP

	PR^a^ (95% CI)	BETA^b^ (95% CI)	BETA^b^ (95% CI)

**Polluting vs clean fuels** ^c^			

Unadjusted	1.08 (0.90, 1.30)	1.58 (–0.87, 4.03)	0.47 (–1.16, 2.10)

Adjusted^c^	0.94 (0.80, 1.10)	–1.16 (–3.18, 0.85)	–0.59 (–2.07, 0.89)

Among males^d^	0.86 (0.66, 1.12)	**–4.80 (–8.24, –1.37)**	–1.36 (–3.96, 1.23)

Among females^d^	0.99 (0.81, 1.20)	0.99 (–1.46, 3.44)	–0.13 (–1.93, 1.67)

**Indoor vs outdoor cooking**			

Unadjusted	**1.13 (1.00, 1.28)**	1.41 (–0.43, 3.24)	0.59 (–0.63, 1.80)

Adjusted^e^	**1.12 (1.00, 1.25)**	**1.67 (0.15, 3.20)**	0.36 (–0.73, 1.45)


a. Prevalence ratio from pooled results of generalized estimating equation Poisson regression models from 10 imputed datasets. b. Beta coefficient from pooled results of general estimating equation linear regression models from 10 imputed datasets. c. Models adjusted for age, sex, poverty, education, food insecurity, household size, and season. d. Models adjusted for age, sex, poverty, education, food insecurity, household size, season, and included an interaction between sex and cooking fuel type. e. Models adjusted for age, sex, poverty, education, food insecurity, household size, cooking fuel type and season.

## Discussion

Using a large cohort in an urban area of Haiti, this study found that 88.2% of participants primarily used polluting fuels for cooking and this was disproportionately greater among those with less economic means. Cooking fuel type was not associated with BP outcomes overall. Indoor cooking was associated with higher HTN prevalence and higher SBP, regardless of cooking fuel type. Sex modified the relationship between cooking fuel type and SBP, where men exposed to polluting compared to clean fuels had lower SBP, but there was no difference in SBP by this exposure among women.

This study suggests that the prevalence of cooking with clean fuels (11.8%) in Port-au-Prince is much lower than in other LMICs, which was 55% in 2021 (1). This is only slightly higher than the 9.8% utilization estimated from the Haiti Demographic and Health Surveys 2016–2017 for urban Haitian areas (12). Among polluting fuels, charcoal use is more widespread, likely due to its lower cost, greater availability, and less equipment needs (35). Eighty percent of charcoal consumed in Port-au-Prince is converted from wood from the southern peninsula and this is the second-largest agricultural sector in the country (35). In other LMICs, biomass fuels like wood are more prevalent (1), but this is generally less accessible in urban areas like Port-au-Prince. There was also low usage of kerosene for cooking (1.6%), similar to that of other developing countries, where less than 1% of households use kerosene for cooking (1).

Multiple barriers exist to the transition to cleaner fuels in Port-au-Prince including cost, irregular access, and safety concerns (11,36). Propane requires higher upfront costs for both the stove and equipment. Cost as a barrier is further supported by our findings that polluting fuel use was more common among those with lower educational status, high food insecurity, and lower income. Further, propane access is routinely disrupted due to conflict and gas inflation, so even those with a propane stove may not use it primarily or may use it in combination with charcoal stoves for foods that require long cooking times to be economically efficient (11). Propane may also be perceived as highly dangerous and responsible for the majority of domestic cooking accidents (causing thermal burns), unlike kerosene, charcoal, and wood (11). It is also noted that solar cooking was not reported among any participants and may be a potential intervention to transition to clean cooking, but high variability in performance due to environmental conditions and also concerns about food safety due to low cooking power are of concern (37).

After adjustment for sociodemographic factors, this study did not find a relationship between cooking fuel type and BP outcomes. Although meta-analyses from observational studies found 50% higher pooled odds of HTN among those using polluting fuels compared to clean fuels, there was high heterogeneity among studies due to differences such as type of stove, ventilation, type of housing, and urban or rural status (18,20). Other recent observational studies also demonstrated mixed results. Epidemiologic studies of Chinese adults from the China Health and Nutrition Survey (CHNS) (38) and the PURE-China study (39) found that polluting fuels were associated with higher HTN compared to clean fuels. Another study of CHNS among rural women only found 0.08 mmHg higher SBP among women <40 years, but no difference among women ≥40 years (40). However, other studies among women in China and rural South Africa showed no association between cooking fuel type and BP outcomes (41–42). Most intervention studies have evaluated improved vs traditional cookstoves, with both arms burning polluting fuels, or evaluated polluting vs clean fuels among pregnant women and these studies were not comparable (19,43–44). There is a need for intervention studies among all females and populations in general in LMICs using polluting vs clean fuels for cooking directly to clarify this relationship.

One reason for null effects may be that cooking fuel is a proxy for economic status. Higher economic status was associated with the use of clean fuels and has also been correlated with other pathways causing elevated BP, such as obesity (45). Another reason may be the study’s inability to accurately classify HAP exposure. Variants to the exposure, like frequency, duration, and intensity of polluting fuel utilization were not measured and may affect BP outcomes differentially. Moreover, a common practice which was also not measured and would also introduce variability in HAP is stove stacking, where individuals use stoves with cheaper, polluting fuels for foods requiring longer cooking time and stoves requiring clean fuels for quicker meals (11). This study also did not distinguish between traditional or improved charcoal stoves, with the latter emitting less pollutants (2). A meta-analysis of interventional studies in LMICs in Asia, Africa and South and Central Americas found a decrease of 2.82 mmHg SBP with improved vs traditional biomass stoves (44). Further, data on outside cooking conditions such as open-air structures adjacent to the home was not recorded and may also affect HAP.

Males had lower SBP in households using polluting vs clean fuel while there was no effect on SBP among women. Reasons for this association are unclear and inconsistent with previous studies. A longitudinal study of Chinese adults did not find an interaction between cooking fuel type and sex on HTN (38). A cross-sectional study of adults in Albania found that women had higher odds of HTN due to polluting vs clean fuels than men (46). Although males typically have lower HTN diagnosis or management (15), the post hoc analysis of those not treated for HTN showed similar findings. It was noted that younger men exposed to polluting fuels had lower SBP than those exposed to clean fuels, but with increasing age, this gap diminished (Supplementary Figure 3). It is also speculated that since homes using clean fuels tended to have higher income, younger men from these homes may also have elevated BP from other risk factors such as alcohol use and smoking. These effects remained in the sensitivity analysis which included smoking but were attenuated.

Indoor cooking was associated with higher HTN prevalence and SBP, despite adjustment for cooking fuel type. Although there is a preference for cooking in outdoor areas to minimize accidents and discomfort caused by smoke, which reduces HAP exposure and mitigates negative health impacts (33), cooking occurred mostly inside the home (16.7%) or inside a separate building (47.3%) for participants using polluting fuels. Use of a separate building may protect other members of the household, but HAP is still exposed to the primary cook. Previous studies have not evaluated indoor vs outdoor cooking but studied indoor polluting fuel vs clean fuel use with inconsistent results (47–48). Cooking location, irrespective of the type of fuel, may be a useful marker of HAP due to the containment of HAP emissions (33). It is noted that even though liquid propane gas (LPG) stoves reduce HAP emissions, they still fail to meet the WHO Air Quality standards for health (2,49). Although a window or door was near the cooking area, ventilation utilization was not recorded. LPG stoves have been promoted as part of the transition to clean fuels in LMICs and are primarily used indoors, but interventions should also consider ventilation mechanisms like chimneys or air filters.

Several considerations should be made when interpreting the results. First, since this was a cross-sectional study, a causal relationship between cooking fuel type and the development of high BP could not be determined. Second, this study was not able to distinguish between commercial cooks (such as food vendors) and domestic cooks, which may also affect both cooking fuel type and cardiovascular health. Third, cooking fuel type was not comprehensively ascertained with respect to frequency, intensity, and duration of exposure, which is important for understanding the cumulative exposure to pollutants emitted and establishing a precise dose-response relationship with elevated BP. Participants may also use both types of cooking fuels and data on combined use was not measured. Fourth, other types of HAP were not controlled for, such as the use of kerosene lamps for lighting or indoor dust exposure. Fifth, although higher HTN prevalence and SBP measures were observed among those who used polluting vs cooking fuels, these differences were too small to be detected statistically from the study sample size. Sixth, occupation and awareness of health implications from cookstoves were not recorded and may confound results. These limitations highlight the need for better HAP exposure assessment in Haiti with larger samples. Lastly, results may not be generalizable to other populations or urban settings outside of Haiti or time periods since access to clean fuels varies regionally and temporally.

Strengths of this study include data from a population of under-surveyed households in urban Port-au-Prince including slum communities highly vulnerable to HAP exposure and rigorous research-grade BP measurements aligned with WHO and AHA guidelines. This study also evaluated cooking location, an understudied, but intervenable reason for HAP exposure. Also, using an interaction to test polluting fuel use by sex allowed insight into effects in both groups.

## Conclusion

In summary, this study found high prevalence of polluting fuel use for cooking in urban Port-au-Prince, and indoor compared to outdoor cooking was associated with higher HTN and SBP. It also identified that those most vulnerable to poverty are the ones most likely to use polluting fuels. This underscores the need for interventions to transition this urban community towards clean cooking fuels to achieve the WHO Sustainable Development Goal 7 target to affordable and clean energy (50). This shift requires the development of an energy infrastructure to make them routinely accessible and affordable. In the meantime, practical and sustainable interventions may include using improved charcoal cookstoves with chimneys, adapting indoor cooking areas to have better ventilation, and potentially cooking outdoors if feasible. Public health programs to promote ventilation use and to provide awareness of the health impacts of cooking with polluting fuels, including elevated blood pressure and cardiovascular diseases can also mitigate this burden.

## Data Accessibility Statement

Researchers who provide a methodologically sound proposal may have access to a subset of deidentified participant data, with specific variables based on the proposal. Proposals should be directed to the principal investigator at mam9365@med.cornell.edu. To gain access, data requestors will need to sign a data access agreement. Data are available following publications through 3 years after publication and will be provided directly from the PI.

## Additional File

The additional file for this article can be found as follows:

10.5334/gh.1405.s1 Supplementary Material. Supplementary figures and table.
