# Supplementary material for: Prevalence of Cooking with Polluting Fuels and Association with Elevated Blood Pressure Among Adults in Port au Prince, Haiti: A Cross-Sectional Analysis
Source: Glob Heart. 2025 Feb 28;20(1):22. doi: 10.5334/gh.1405 (PMC11869832; doi:10.5334/gh.1405)
Supplement: Supplementary Material. — Supplementary figures and table. [file gh-20-1-1405-s1.pdf]

Supplementary Material

Supplementary Figure 1. Study flow diagram.

Supplementary Figure 2. Directed acyclical graph of relationship between polluting fuel use and blood pressure.

Supplementary Figure 3. Distribution of systolic and diastolic blood pressure by sex and cooking fuel type.

Supplementary Table 1. Results from main and post hoc sensitivity analyses.

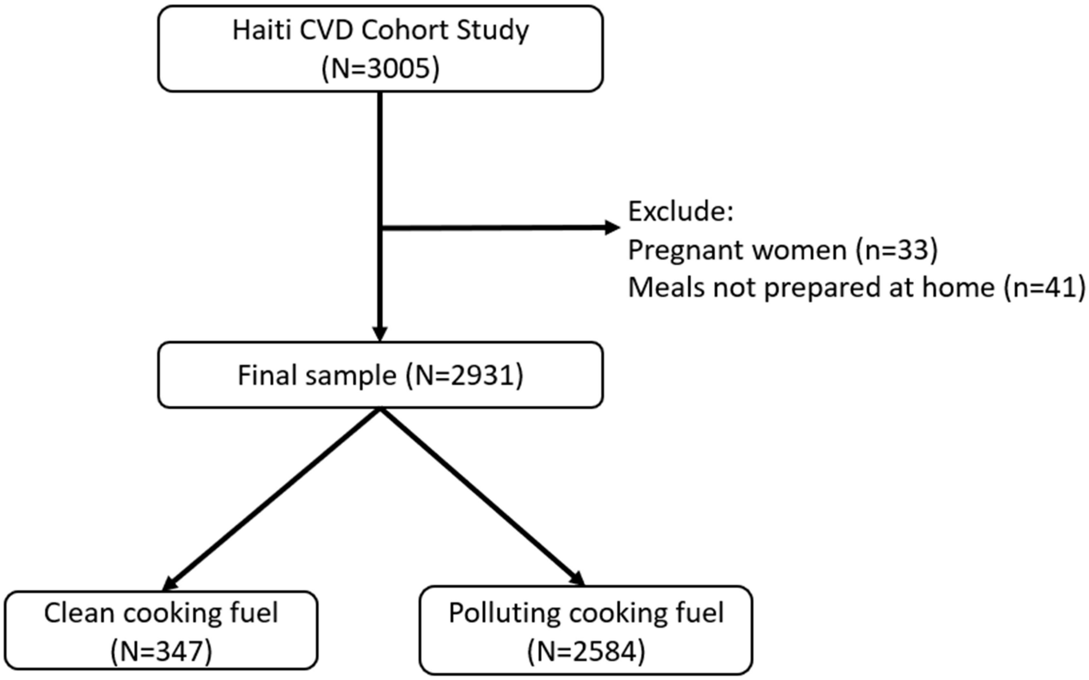

Supplementary Figure 1. Study flow diagram.

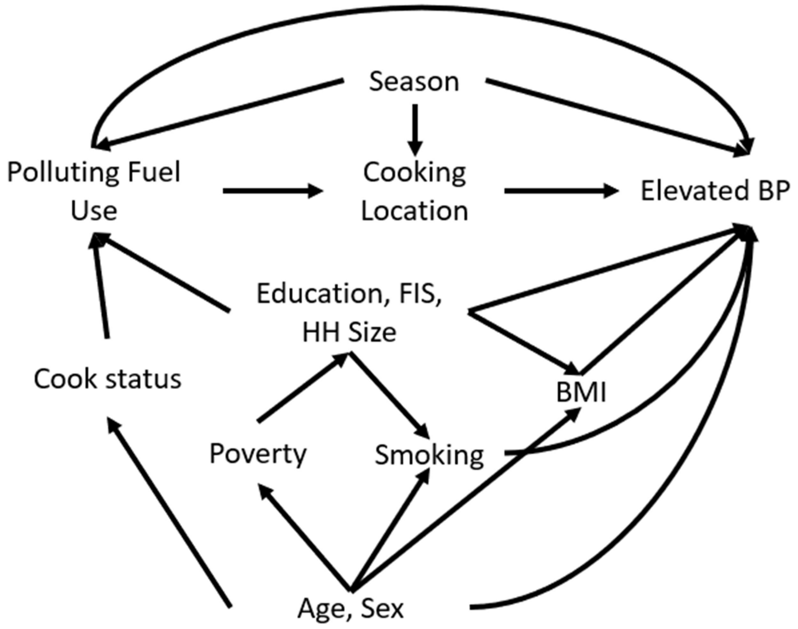

Supplementary Figure 2. Directed acyclical graph of relationship between polluting fuel use and blood pressure.

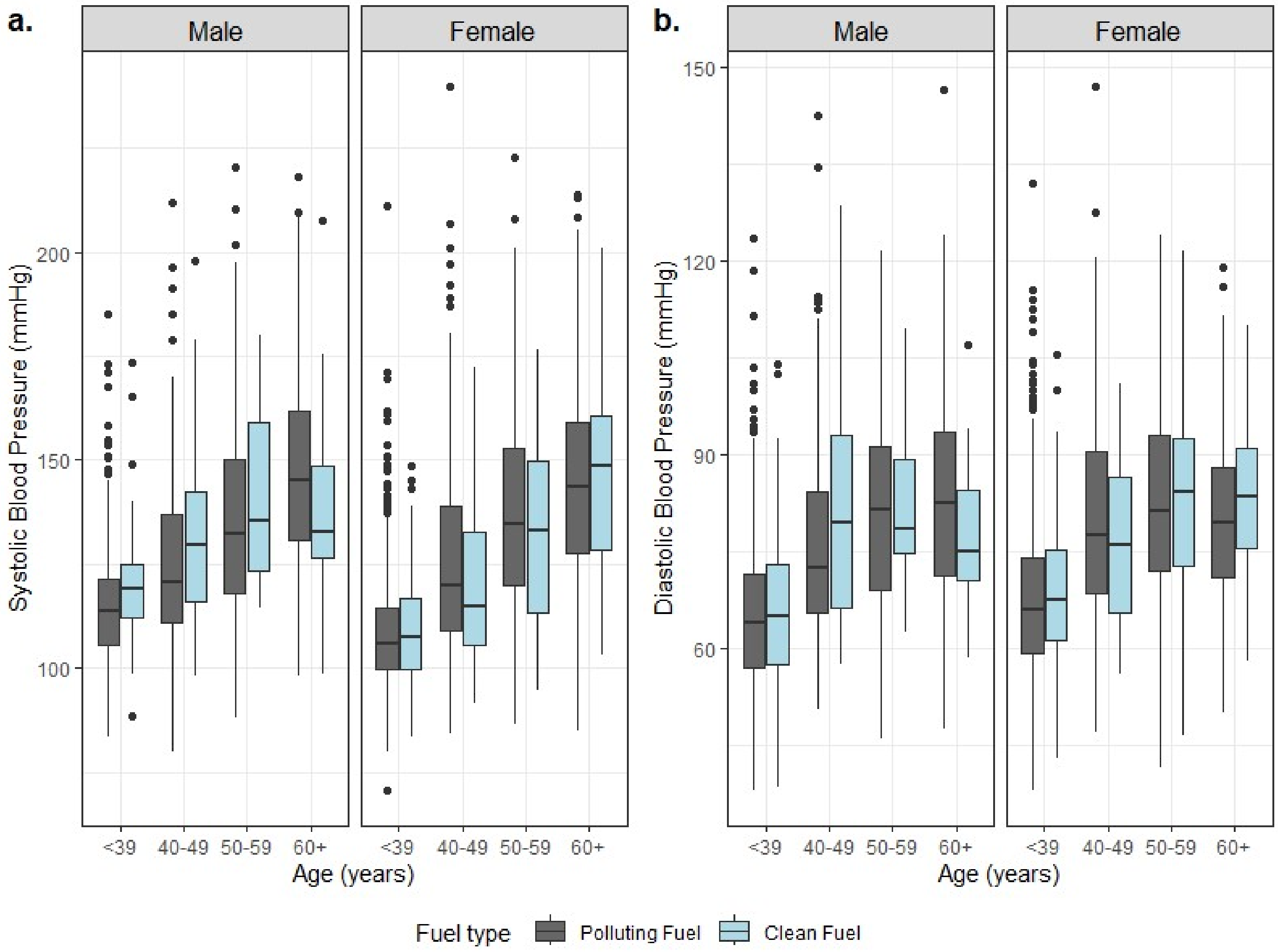

Supplementary Figure 3. Distribution of (a) systolic and (b) diastolic blood pressure by sex and cooking fuel type.

Model	Hypertension	SBP	DBP
	PR[a] (95% CI)	Beta[b] (95% CI)	Beta[b] (95% CI)
**Polluting vs clean fuels**			
**Overall[c]**			
Main	0.94 (0.80, 1.10)	-1.16 (-3.18, 0.85)	-0.59 (-2.07, 0.89)
S1: Exclude those on BP medications	0.97 (0.75, 1.26)	-0.96 (-2.98, 1.06)	-0.27 (-1.83, 1.28)
S2: Include cooks only	0.93 (0.74, 1.18)	-0.39 (-3.50, 2.72)	-0.69 (-2.95, 1.56)
S3: Include >/=35 yr olds	0.91 (0.78, 1.06)	-0.82 (-3.92, 2.28)	-1.04 (-3.13, 1.06)
S4: Include smoking and BMI status	1.00 (0.86, 1.18)	-0.31 (-2.31, 1.68)	0.27 (-1.17, 1.72)
**Among females[d]**			
Main	0.99 (0.81, 1.20)	0.99 (-1.46, 3.44)	-0.13 (-1.93, 1.67)
S1: Exclude those on BP medications	1.06 (0.76, 1.48)	1.27 (-1.22, 3.77)	0.38 (-1.48, 2.24)
S3: Include >/=35 yr olds	0.94 (0.78, 1.14)	1.24 (-2.32, 4.80)	-0.49 (-2.85, 1.88)
S4: Include smoking and BMI status	1.05 (0.86, 1.27)	1.80 (-0.62, 4.23)	0.72 (-1.04, 2.47)
**Among Males[d]**			
Main	0.86 (0.66, 1.12)	-4.80 (-8.24, -1.37)	-1.36 (-3.96, 1.23)
S1: Exclude those on BP medications	0.84 (0.56, 1.25)	-4.58 (-7.93, -1.23)	-1.33 (-4.05, 1.40)
S3: Include >/=35 yr olds	0.86 (0.66, 1.11)	-4.73 (-10.63, 1.17)	-2.08 (-6.01, 1.86)
S4: Include smoking and BMI status	0.93 (0.72, 1.20)	-3.89 (-7.24, -0.54)	-0.48 (-2.99, 2.04)
**Indoor vs outdoor cooking[e]**			
Main	1.12 (1.00, 1.25)	1.67 (0.15, 3.20)	0.36 (-0.73, 1.45)
S1: Exclude those on BP medications	1.13 (0.97, 1.32)	1.41 (-0.09, 2.91)	0.45 (-0.64, 1.55)
S2: Include cooks only	1.15 (0.96, 1.37)	1.19 (-1.23, 3.61)	-0.35 (-2.07, 1.37)

S3: Include >/=35 yr olds	1.14 (1.02, 1.27)	2.58 (0.32, 4.84)	0.53 (-0.99, 2.04)
S4: Include smoking and BMI status	1.12 (1.00, 1.25)	1.55 (0.03, 3.06)	0.25 (-0.82, 1.32)

Supplementary Table 1. Results from main and post-hoc sensitivity analyses. PR=prevalence ratio, CI=confidence interval, SBP=systolic blood pressure, DBP=diastolic blood pressure.
a. Prevalence ratio from pooled results of generalized estimating equation Poisson regression models from 10 imputed datasets.
b. Beta coefficient from pooled results of general estimating equation linear regression models from 10 imputed datasets.
c. Models adjusted for age, sex, poverty, education, food insecurity, household size, and season.
d. Models adjusted for age, sex, poverty, education, food insecurity, household size, season, and included an interaction between sex and cooking fuel type.
e. Models adjusted for age, sex, poverty, education, food insecurity, household size, cooking fuel type and season.
